# Incorporating Culture into The Treatment of Substance Use Disorder: A Narrative Review

**DOI:** 10.1192/j.eurpsy.2023.1397

**Published:** 2023-07-19

**Authors:** O. Nombora, A. Certo, B. F. Silva, Â. Venâncio

**Affiliations:** Psychiatry Department, Centro Hospitalar Vila Nova de Gaia/Espinho, EPE, Vila Nova de Gaia, Portugal

## Abstract

**Introduction:**

Culture is defined by the shared beliefs, attitudes, values, and practices of a particular group of people which can influence their behaviour and social interactions, including the use of substances.

**Objectives:**

The aim of this review is to identify the evidence of cultural competence in the treatment of people with substance use disorder (SUD) and encourage the professionals and organizations to take cultural context into account.

**Methods:**

Narrative review about the topic, using PubMed/Medline database. MeSH terms: *“culture”, “cultural competence”, “addictions”, “substance use disorder”.*

**Results:**

Studies show that culture can either be a catalyst for SUD or play a protective role. However, other factors may also play a large role in client’s response substance use and the development of SUD. Acculturation and generational differences can also impact SUD treatment, especially when intergenerational conflict causes stress that leads individuals to engage in risky behaviours. Thus, treatment for SUD has to be sensitive to cultural differences and professionals should provide culturally based approaches. Culturally targeted practices have been linked to greater outcomes, better therapeutic alliance, less dropouts and consequent increased retention in the treatment. These practices include matching clinicians and clients on linguistic and cultural backgrounds as well as being mindful of the impact of culture on client’s experience of SUD. Providing therapy and materials in the client’s language, knowledge, understanding and appreciation for cultural perspectives, involving the family and community and training therapists, are some of culturally competent practices used. These strategies involve knowledge, creativity, and experience.

**Conclusions:**

Cultural competence seems to be a valuable tool for healthcare professionals working in a multicultural context, particularly with people with SUD. Unfortunately, the lack of supporting evidence limits the validity of any particular model of cultural competence. Future methodologically research is necessary in order to provide quality cultural competence models for people with SUD.

**Disclosure of Interest:**

None Declared

